# Hexa­kis­(*N*,*N*′-dimethyl­thio­urea-κ*S*)nickel(II) nitrate

**DOI:** 10.1107/S1600536810040031

**Published:** 2010-10-13

**Authors:** Iram Asif, Rashid Mahmood, Helen Stoeckli-Evans, Muhammad Mateen, Saeed Ahmad

**Affiliations:** a Department of Chemistry, University of Engineering and Technology, Lahore 54890, Pakistan; b Division of Science and Technology, University of Education, Township, Lahore, Pakistan; c Institute of Physics, University of Neuchâtel, rue Emile-Argand 11, CH-2000 Neuchâtel, Switzerland

## Abstract

The title complex salt, [Ni(C_3_H_8_N_2_S)_6_](NO_3_)_2_, consists of an [Ni(Dmtu)_6_]^2+^ (Dmtu is *N*,*N*′-dimethyl­thio­urea) dication and two nitrate counter-anions. The Ni^II^ atom (site symmetry 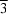) is coordinated by the S atoms of six Dmtu ligands within a slightly distorted octa­hedral environment. The crystal structure is characterized by weak intra­molecular N—H⋯S inter­actions and by inter­molecular N—H⋯O hydrogen bonds involving the nitrate anion (site symmetry 3.). These inter­molecular inter­actions lead to the formation of two-dimensional networks lying parallel to the *ab* plane. The networks are linked *via* non-classical inter­molecular C—H⋯O hydrogen bonds, forming a three-dimensional arrangement.

## Related literature

For background to nickel(II) complexes of thio­urea and its derivatives, see: Ambujam *et al.* (2006 ▶); Basso *et al.* (1969 ▶); Bentley & Waters (1974 ▶); Chiesi *et al.* (1971 ▶); Crane & Herod (2004 ▶); Eaton & Zaw (1975 ▶); El-Bahy *et al.* (2003 ▶); Figgis & Reynolds (1986 ▶); Monim-ul-Mehboob *et al.* (2010 ▶); Sonar *et al.* (1979 ▶); Weininger *et al.* (1969 ▶); Weininger & Amma (1976 ▶). For the crystal structures of similar nickel(II) complexes, see: Bentley & Waters (1974 ▶); El-Bahy *et al.* (2003 ▶); Monim-ul-Mehboob *et al.* (2010 ▶); Weininger *et al.* (1969 ▶).
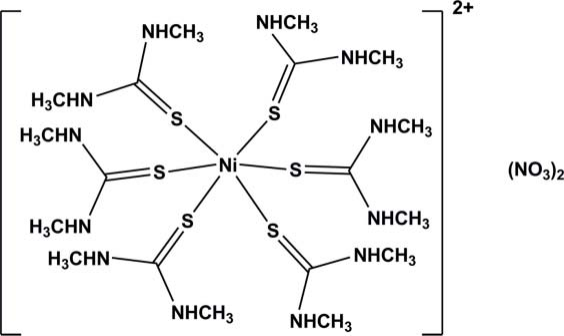


## Experimental

### 

#### Crystal data

[Ni(C_3_H_8_N_2_S)_6_](NO_3_)_2_
*M* *_r_* = 807.77
Trigonal, 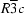

*a* = 13.7166 (10) Å
*c* = 35.332 (3) Å
*V* = 5756.9 (8) Å^3^
*Z* = 6
Mo *K*α radiation
μ = 0.88 mm^−1^
*T* = 223 K
0.30 × 0.26 × 0.24 mm

#### Data collection

Stoe IPDS 2 diffractometer
Absorption correction: multi-scan (MULscanABS; Spek, 2009 ▶) *T*_min_ = 0.963, *T*_max_ = 1.000
3491 measured reflections
1199 independent reflections
851 reflections with *I* > 2σ(*I*)
*R*_int_ = 0.028

#### Refinement

*R*[*F* ^2^ > 2σ(*F* ^2^)] = 0.029
*wR*(*F* ^2^) = 0.056
*S* = 1.00
1199 reflections
79 parameters
2 restraints
H atoms treated by a mixture of independent and constrained refinement
Δρ_max_ = 0.17 e Å^−3^
Δρ_min_ = −0.18 e Å^−3^

### 

Data collection: *X-AREA* (Stoe & Cie, 2009 ▶); cell refinement: *X-AREA*; data reduction: *X-RED32* (Stoe & Cie, 2009 ▶); program(s) used to solve structure: *SHELXS97* (Sheldrick, 2008 ▶); program(s) used to refine structure: *SHELXL97* (Sheldrick, 2008 ▶); molecular graphics: *PLATON* (Spek, 2009 ▶); software used to prepare material for publication: *SHELXL97*, *PLATON* and *publCIF* (Westrip, 2010 ▶).

## Supplementary Material

Crystal structure: contains datablocks I, global. DOI: 10.1107/S1600536810040031/wm2412sup1.cif

Structure factors: contains datablocks I. DOI: 10.1107/S1600536810040031/wm2412Isup2.hkl

Additional supplementary materials:  crystallographic information; 3D view; checkCIF report

## Figures and Tables

**Table 1:** Hydrogen-bond geometry (Å, °)

*D*—H⋯*A*	*D*—H	H⋯*A*	*D*⋯*A*	*D*—H⋯*A*
N1—H1*N*⋯S1^i^	0.86 (2)	2.520 (19)	3.367 (2)	168.6 (17)
N2—H2*N*⋯O1^ii^	0.83 (2)	2.14 (2)	2.947 (3)	163.4 (18)
C3—H3*B*⋯O1^iii^	0.97	2.41	3.180 (3)	136

Symmetry codes: (i) 
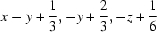
; (ii) 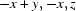; (iii) 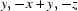.

**Fig. 1.**
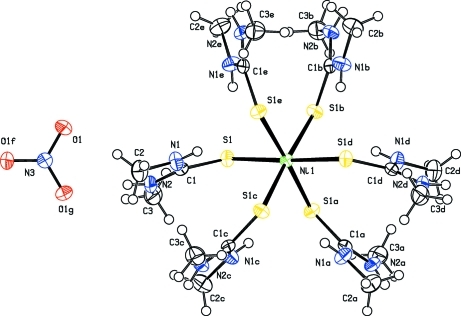
The molecular structure of the title compound with the atomic numbering scheme. Displacement ellipsoids are drawn at the 50% probability level [Only one of the nitrate anions is shown; Symmetry codes: a = 1 - y,x-y,z; b = 1 - x + y,1 - x,z; c = 1/3 + y,-1/3 + x,1/6 - z; d = 4/3 - x,2/3 - x + y,1/6 - z; e = 1/3 + x-y,2/3 - y,1/6 - z; f = -y,x-y,z; g = -x + y,-x,z].

**Fig. 2.**
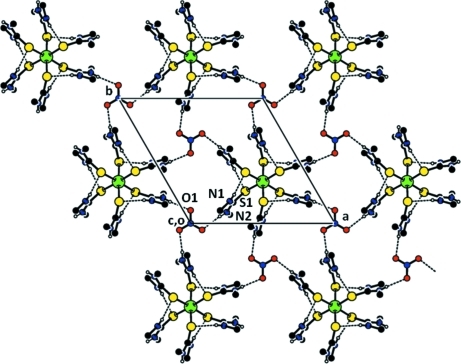
The crystal packing of the title compound viewed along the c axis (the N—H···O and N—H···S hydrogen bonds are shown as dashed lines - see Table 1 for details; H-atoms not involved in hydrogen bonding have been omitted for clarity).

## supplementary crystallographic information

### Comment

Several studies have been focused on the synthesis and structural characterization of nickel(II) complexes with thiourea type ligands. These studies showed that nickel(II) can adopt a variety of coordination geometries (octahedral, tetragonal, square-planar and tetrahedral) both in the solid state and in solution, which were prepared by varying the ligands or the anions (Ambujam *et al.*, 2006; Bentley *et al.*, 1974; Chiesi *et al.*, 1971; Eaton & Zaw, 1975; El-Bahy *et al.*, 2003; Figgis & Reynolds, 1986; Monim-ul-Mehboob *et al.*, 2010; Sonar *et al.*, 1979; Weininger *et al.* 1969, Weininger & Amma, 1976). When the anion is chloride, bromide or iodide, the predominant coordination about the nickel(II) atom in the crystalline solid state is tetragonal with the halide anions in the apical positions, leading to [Ni*L*_4_]*X*_2_ complexes (Ambujam *et al.*, 2006; Chiesi *et al.*, 1971; Crane *et al.*, 2004; Figgis & Reynolds, 1986; Weininger & Amma, 1976), although [Ni*L*_6_]*X*_2_ complexes are also formed (El-Bahy *et al.*, 2003; Weininger *et al.*, 1969). The formation (in the solid state) of the octahedral species Ni*L*_6_^2+^ is ascribed to crystal packing forces and extensive hydrogen bonding (Ambujam *et al.*, 2006; El-Bahy *et al.*, 2003; Monim-ul-Mehboob *et al.*, 2010; Weininger *et al.*, 1969). The coordination of nickel(II) in nitrate and the perchlorate salts is generally homoleptic octahedral in the solid state (Bentley *et al.*, 1974; Monim-ul-Mehboob *et al.*, 2010), but also can give such species as [Ni*L*_2_(NO_3_)_2_] (Basso *et al.*, 1969). We have recently reported on the crystal structure of a thiourea (Tu) complex of nickel(II) nitrate, [Ni(Tu)_6_](NO_3_)_2_ (Monim-ul-Mehboob *et al.*, 2010). Herein, we report on the crystal structure of the title nickel(II) nitrate complex of dimethylthiourea, [Ni(Dmtu)_6_](NO_3_)_2_.

The molecular structure of the title complex is illustrated in Fig. 1. It is ionic and consists of a [Ni(Dmtu)_6_]^2+^ cationic unit (site symmetry 3) and two nitrate counter ions (site symmetry 3.). Atom Ni1 assumes a slightly distorted octahedral geometry, due to coordination with six sulfur atoms of the Dmtu ligands. In the cation there are weak N—H···S interactions linking adjacent ligand molecules (Table 1). The values of the bond lengths and bond angles observed in the title complex are comparable to those reported for related complexes (Ambujam *et al.*, 2006; El-Bahy *et al.*, 2003; Monim-ul-Mehboob *et al.*, 2010; Weininger *et al.*, 1969). In the only previously reported nickel(II) complex of *N*,*N*'-dimethylthiourea, [Ni(Dmtu)_4_]Br_2_ (Weininger & Amma, 1976), the nickel(II) atom is 4-coordinate, while in the title complex having the same ligand the nickel(II) atom is 6-coordinate, suggesting that in the presence of nitrate an octahedral coordination is preferred.

In the crystal of the title compound the [Ni(Dmtu)_6_]^+2^ cations and the NO_3_^-^ ions are connected *via* N—H···O hydrogen bonds (Table 1) to form two-dimensional networks lying parallel to the *ab*-plane (Fig. 2). These two-dimensional sheets are linked *via* C—H···O hydrogen bonds (Table 1), resulting in the formation of a three-dimensional network.

### Experimental

The title compound was prepared by adding 2 equivalents of *N*,*N*'-dimethylthiourea in 15 ml methanol to 0.29 g (1 mmol) of nickel(II) nitrate hexahydrate in 10 ml methanol. After stirring the mixture for 30 min the solution was filtered. The filtrate on slow evaporation yielded pale-green crystals, suitable for X-ray diffraction analysis.

### Refinement

The NH H-atoms were located in difference electron-density maps. In the final cycles of least-squares refinement they was refined with a distance restraint of N—H = 0.87 (2) Å. The C-bound H-atoms were included in calculated positions and treated as riding atoms: C—H = 0.97 Å for CH_3_ H-atoms, with *U*_iso_(H) = 1.5*U*_eq_ (parent C-atom).

### Crystal data

**Table:**

[Ni(C_3_H_8_N_2_S)_6_](NO_3_)_2_	*D*_x_ = 1.398 Mg m^−^^3^
*M**_r_* = 807.77	Mo *K*α radiation, λ = 0.71073 Å
Trigonal, *R*3*c*	Cell parameters from 2717 reflections
Hall symbol: -R 3 2"c	θ = 2.9–26.1°
*a* = 13.7166 (10) Å	µ = 0.88 mm^−^^1^
*c* = 35.332 (3) Å	*T* = 223 K
*V* = 5756.9 (8) Å^3^	Block, pale green
*Z* = 6	0.30 × 0.26 × 0.24 mm
*F*(000) = 2556	

### Data collection

**Table:**

Stoe IPDS 2 diffractometer	1199 independent reflections
Radiation source: fine-focus sealed tube	851 reflections with *I* > 2σ(*I*)
graphite	*R*_int_ = 0.028
φ + ω scans	θ_max_ = 25.6°, θ_min_ = 2.9°
Absorption correction: multi-scan (MULscanABS; Spek, 2009)	*h* = −4→14
*T*_min_ = 0.963, *T*_max_ = 1.000	*k* = −16→9
3491 measured reflections	*l* = −42→40

### Refinement

**Table:**

Refinement on *F*^2^	Primary atom site location: structure-invariant direct methods
Least-squares matrix: full	Secondary atom site location: difference Fourier map
*R*[*F*^2^ > 2σ(*F*^2^)] = 0.029	Hydrogen site location: inferred from neighbouring sites
*wR*(*F*^2^) = 0.056	H atoms treated by a mixture of independent and constrained refinement
*S* = 1.00	*w* = 1/[σ^2^(*F*_o_^2^) + (0.0271*P*)^2^] where *P* = (*F*_o_^2^ + 2*F*_c_^2^)/3
1199 reflections	(Δ/σ)_max_ = 0.001
79 parameters	Δρ_max_ = 0.17 e Å^−^^3^
2 restraints	Δρ_min_ = −0.18 e Å^−^^3^

### Special details

**Table:**

Geometry. Bond distances, angles *etc*. have been calculated using the rounded fractional coordinates. All su's are estimated from the variances of the (full) variance-covariance matrix. The cell e.s.d.'s are taken into account in the estimation of distances, angles and torsion angles
Refinement. Refinement of *F*^2^ against ALL reflections. The weighted *R*-factor *wR* and goodness of fit *S* are based on *F*^2^, conventional *R*-factors *R* are based on *F*, with *F* set to zero for negative *F*^2^. The threshold expression of *F*^2^ > σ(*F*^2^) is used only for calculating *R*-factors(gt) *etc*. and is not relevant to the choice of reflections for refinement. *R*-factors based on *F*^2^ are statistically about twice as large as those based on *F*, and *R*- factors based on ALL data will be even larger.

### Fractional atomic coordinates and isotropic or equivalent isotropic displacement parameters (Å^2^)

**Table:**

	*x*	*y*	*z*	*U*_iso_*/*U*_eq_	
Ni1	0.66667	0.33333	0.08333	0.0208 (1)	
S1	0.50780 (4)	0.25803 (5)	0.03726 (1)	0.0262 (1)	
N1	0.35924 (14)	0.17766 (18)	0.09365 (4)	0.0319 (5)	
N2	0.29514 (15)	0.09027 (15)	0.03650 (5)	0.0295 (5)	
C1	0.37831 (17)	0.16823 (15)	0.05723 (5)	0.0246 (6)	
C2	0.25557 (19)	0.10275 (19)	0.11355 (6)	0.0399 (7)	
C3	0.3066 (2)	0.0703 (2)	−0.00341 (6)	0.0409 (8)	
O1	0.05182 (15)	0.10490 (12)	0.05290 (4)	0.0449 (5)	
N3	0.00000	0.00000	0.05255 (7)	0.0300 (6)	
H1N	0.4165 (16)	0.2300 (15)	0.1053 (5)	0.029 (6)*	
H2A	0.19490	0.11240	0.10370	0.0600*	
H2B	0.26520	0.12040	0.14040	0.0600*	
H2C	0.23750	0.02540	0.10990	0.0600*	
H2N	0.2303 (14)	0.0569 (17)	0.0455 (5)	0.021 (5)*	
H3A	0.34410	0.14160	−0.01680	0.0610*	
H3B	0.23250	0.02290	−0.01430	0.0610*	
H3C	0.35070	0.03320	−0.00570	0.0610*	

### Atomic displacement parameters (Å^2^)

**Table:**

	*U*^11^	*U*^22^	*U*^33^	*U*^12^	*U*^13^	*U*^23^
Ni1	0.0195 (2)	0.0195 (2)	0.0232 (2)	0.0098 (1)	0.0000	0.0000
S1	0.0203 (2)	0.0277 (3)	0.0272 (2)	0.0095 (3)	−0.0019 (2)	−0.0009 (3)
N1	0.0223 (8)	0.0328 (11)	0.0325 (7)	0.0078 (10)	−0.0009 (6)	−0.0031 (9)
N2	0.0180 (9)	0.0281 (10)	0.0382 (8)	0.0084 (8)	−0.0036 (8)	−0.0040 (8)
C1	0.0218 (10)	0.0222 (11)	0.0336 (9)	0.0138 (8)	−0.0037 (7)	0.0011 (7)
C2	0.0299 (12)	0.0453 (15)	0.0373 (10)	0.0133 (11)	0.0033 (9)	0.0028 (9)
C3	0.0376 (14)	0.0439 (15)	0.0398 (11)	0.0193 (12)	−0.0136 (10)	−0.0131 (10)
O1	0.0306 (10)	0.0206 (7)	0.0794 (10)	0.0097 (9)	0.0077 (10)	0.0079 (7)
N3	0.0261 (9)	0.0261 (9)	0.0379 (13)	0.0131 (5)	0.0000	0.0000

### Geometric parameters (Å, °)

**Table:**

Ni1—S1	2.4929 (6)	N2—C3	1.460 (3)
Ni1—S1^i^	2.4929 (7)	N2—C1	1.327 (3)
Ni1—S1^ii^	2.4929 (5)	N1—H1N	0.86 (2)
Ni1—S1^iii^	2.4929 (5)	N2—H2N	0.83 (2)
Ni1—S1^iv^	2.4929 (6)	C2—H2B	0.9700
Ni1—S1^v^	2.4929 (7)	C2—H2C	0.9700
S1—C1	1.727 (2)	C2—H2A	0.9700
O1—N3	1.2462 (14)	C3—H3B	0.9700
N1—C1	1.332 (2)	C3—H3C	0.9700
N1—C2	1.453 (3)	C3—H3A	0.9700
			
S1—Ni1—S1^i^	81.98 (2)	C1—N2—H2N	118.9 (13)
S1—Ni1—S1^ii^	81.98 (2)	C3—N2—H2N	116.8 (13)
S1—Ni1—S1^iii^	99.78 (2)	O1—N3—O1^vi^	119.99 (14)
S1—Ni1—S1^iv^	177.39 (2)	O1^vii^—N3—O1^vi^	119.99 (14)
S1—Ni1—S1^v^	96.33 (2)	O1—N3—O1^vii^	119.99 (14)
S1^i^—Ni1—S1^ii^	81.98 (2)	N1—C1—N2	118.7 (2)
S1^i^—Ni1—S1^iii^	96.34 (2)	S1—C1—N2	120.83 (15)
S1^i^—Ni1—S1^iv^	99.78 (2)	S1—C1—N1	120.48 (16)
S1^i^—Ni1—S1^v^	177.40 (2)	N1—C2—H2B	109.00
S1^ii^—Ni1—S1^iii^	177.40 (3)	H2A—C2—H2C	110.00
S1^ii^—Ni1—S1^iv^	96.33 (2)	N1—C2—H2C	109.00
S1^ii^—Ni1—S1^v^	99.78 (2)	H2A—C2—H2B	110.00
S1^iii^—Ni1—S1^iv^	81.98 (2)	N1—C2—H2A	109.00
S1^iii^—Ni1—S1^v^	81.98 (2)	H2B—C2—H2C	109.00
S1^iv^—Ni1—S1^v^	81.97 (2)	N2—C3—H3C	109.00
Ni1—S1—C1	113.77 (7)	H3A—C3—H3C	109.00
C1—N1—C2	124.68 (19)	H3B—C3—H3C	110.00
C1—N2—C3	123.7 (2)	H3A—C3—H3B	109.00
C1—N1—H1N	113.7 (13)	N2—C3—H3A	110.00
C2—N1—H1N	121.5 (13)	N2—C3—H3B	109.00
			
S1^i^—Ni1—S1—C1	124.24 (8)	Ni1—S1—C1—N2	−154.41 (17)
S1^ii^—Ni1—S1—C1	−152.79 (8)	C2—N1—C1—S1	−176.58 (19)
S1^iii^—Ni1—S1—C1	29.15 (8)	C2—N1—C1—N2	4.9 (4)
S1^v^—Ni1—S1—C1	−53.76 (8)	C3—N2—C1—S1	2.9 (3)
Ni1—S1—C1—N1	27.1 (2)	C3—N2—C1—N1	−178.6 (2)

Symmetry codes: (i) −*y*+1, *x*−*y*, *z*; (ii) −*x*+*y*+1, −*x*+1, *z*; (iii) *y*+1/3, *x*−1/3, −*z*+1/6; (iv) −*x*+4/3, −*x*+*y*+2/3, −*z*+1/6; (v) *x*−*y*+1/3, −*y*+2/3, −*z*+1/6; (vi) −*x*+*y*, −*x*, *z*; (vii) −*y*, *x*−*y*, *z*.

### Hydrogen-bond geometry (Å, °)

**Table:**

*D*—H···*A*	*D*—H	H···*A*	*D*···*A*	*D*—H···*A*
N1—H1N···S1^v^	0.86 (2)	2.520 (19)	3.367 (2)	168.6 (17)
N2—H2N···O1^vi^	0.83 (2)	2.14 (2)	2.947 (3)	163.4 (18)
C3—H3B···O1^viii^	0.97	2.41	3.180 (3)	136

Symmetry codes: (v) *x*−*y*+1/3, −*y*+2/3, −*z*+1/6; (vi) −*x*+*y*, −*x*, *z*; (viii) *y*, −*x*+*y*, −*z*.
